# Tridimensional finite element analysis of teeth movement induced by different headgear forces

**DOI:** 10.1186/s40510-016-0130-4

**Published:** 2016-06-06

**Authors:** Ivan Toshio Maruo, Hiroshi Maruo, Armando Yukio Saga, Dauro Douglas de Oliveira, Marco André Argenta, Orlando Motohiro Tanaka

**Affiliations:** Orthodontic Graduation Program, Brazilian Dental Association (ABO) and Pontifícia Universidade Católica do Paraná, Curitiba, Paraná Brazil; Orthodontic Graduation Program, Brazilian Dental Association (ABO), Ponta Grossa, Paraná Brazil; Orthodontic Graduation Program, Pontifical Catholic University of Minas Gerais, Belo Horizonte, Minas Gerais Brazil; Post Graduation Program in Numerical Methods in Engineering, Federal University of Paraná, Curitiba, Brazil; Graduate Program in Orthodontics, Pontifícia Universidade Católica do Paraná, School of Life Sciences, R. Imaculada Conceição, 1155 Curitiba, Brazil; Post-Doctoral fellowship at The Center for Advanced Dental Education, Saint Louis University, Saint Louis, MO USA

**Keywords:** Extraoral traction appliances, Finite element analysis, Tooth movement

## Abstract

**Background:**

This study aimed to simulate the actions of low-pull (LP), high-pull (HP), and combined pull (CP) headgears (HGs) and to analyze tooth movement tendencies through finite element analysis.

**Methods:**

Tomographic slices of a human maxilla with complete permanent dentition were processed by reconstruction software, and the triangular surface mesh was converted into non-uniform rational B-spline (NURBS) curves. An HG facial bow was also modulated in 3D. The teeth and bone were considered to have isotropic and linear behavior, whereas the periodontal ligament was considered to have non-linear and hyperelastic behavior. Data regarding the application points, directions and magnitudes of forces were obtained from the literature and from a dolichofacial patient with class II, division 1 malocclusion, who was treated with a CP HG.

**Results:**

The CP HG promoted 37.1 to 41.1 %, and the HP HG promoted 19.1 to 31.9 % of LP distalization. The HP HG presented the highest intrusion, and the LP HG presented the highest extrusion of the first molar. The LP HG contracted the distal side, and the HP and CP HGs contracted the lingual and distobuccal roots of the second molar to a lesser degree.

**Conclusions:**

The LP HG promotes the greatest distalization, followed by the CP and HP HGs; the LP HG causes greater extrusion of the first molar, and the HP HG causes greater intrusion of the first molar. The LP HG causes greater contraction of the second molar than the HP HG.

## Background

Although dental distalizers and skeletal temporary anchorage devices are available, the headgear (HG) appliance is an effective treatment for class II malocclusions in growing patients [1] and is utilized by more than half of orthodontists [2].

HG can be utilized with low (or cervical) pull (LP) [3], high (or parietal) pull (HP) [4], or combined (cervical and parietal) pull (CP) [5]. While unilateral forces of 250 to 500 gf promote orthopedic-orthodontic effects (i.e., restrain maxillary growth), weaker forces induce exclusively orthodontic effects [6].

The concepts of applied mechanics can be used to study dental movement induced by HGs [7]. However, as this methodology does not account for the biological properties of the periodontal ligament, teeth and bone, its results are limited.

Cephalometric clinical studies [4, 5, 8], which compare initial and final results and facilitate patient follow-up using medical records, are useful but also have limitations. As their samples consist of growing patients, it is difficult to isolate appliance effects from inherent craniofacial growth, as well as to distinguish orthopedic from orthodontic effects. In addition, there is the possibility of error when performing radiographs, cephalometric tracings, and measurements [9].

In an attempt to overcome these limitations, finite element analysis (FEA) may be used. FEA is used to predict stress effects on mini-implants and surrounding bone [10], to determine the stresses in bracket-cement-enamel systems [11], to assess the effects of rapid maxillary expansion on the airway flow rate [12], and to evaluate the effects of orthodontic devices on tooth displacement trends. FEA also [13] provides information about the distributions and vector directions of the principal stresses on the periodontal ligament [14–16] and along bone structures [17–19].

By applying FEA, it is possible to shape and analyze dentomaxillofacial structures by dividing complex structures into smaller sections called elements, in which physical properties are applied to dictate an object’s response to an external stimulus, such as orthodontic force [20].

Although the orthopedic effects of different pulls of HGs have been studied through FEA [21], their orthodontic effects in complete permanent dentition have not received the same attention.

Thus, the objectives of this study were to simulate the actions of LP, HP, and CB HGs and to analyze teeth movement tendencies using FEA.

## Methods

### Teeth and maxilla modeling

This study was approved by the Research Ethics Committee of Pontifical Catholic University of Paraná. A dry human skull with complete permanent dentition (except for the absence of third molars) and without caries or restorations was obtained from the Anatomy Department of (omitted). To construct the geometry, the maxilla region below the palatine plane and anterior to the pterygopalatine fossae of the skull was precisely reconstructed based on tomographic images obtained by cone beam computerized tomography (Classic i-CAT®, Imaging Sciences, Hatfield, PA) at 120 kVp, 0.5 mm nominal focal point size, 14 bits of grayscale dynamic range, and 0.4 mm voxel size, producing 256 slices with 0.25 mm thickness, and converted into exportable DICOM files.

Tomographic slices were processed by digital technology, delimiting cortical and cancellous bone and the enamel, dentin, and pulp layers. These limits were utilized to generate 3D geometry by using an assisted design program (Simpleware®, Innovation Centre, Exeter, UK). The generated solid was exported to the Solidworks® program (Dessault Systèmes Solidworks Corp., Concord, MA) to convert the surface mesh into non-uniform rational B-spline (NURBS) curves. This conversion allowed better manipulation and control of generated curves and surfaces. These data were exported to ANSYS® v. 12.1 (Swanson Analysis System Inc., Canonsburg, PA).

The centers of resistance of the first and second molars were assumed to be at the trifurcation of the roots [7]. The centers of resistance of the other teeth were assumed to be at a point 0.4 times the distance from the alveolar crest to the apex [7].

Each tooth was divided into pulp, dentine, and enamel, and the alveolar bone was divided into cortical and cancellous bone. The periodontal ligament (PDL) was simulated as a 0.25-mm layer around the tooth root [22].

The mechanical properties of the teeth and bone were assumed to be homogeneous, isotropic, and linearly elastic, with a specific Young’s modulus and Poisson’s ratio (Table 1). Dental pulp was disregarded in the equation due to its irrelevant stiffness in comparison to the other model components [23]. The typical nonlinear and hyperelastic mechanical behaviors of the PDL were represented by the constitutive model of Natali et al. [24].

**Table 1:** Mechanical properties of the teeth and bone, utilized in the model

Material	Young’s modulus (MPa)	Poisson’s ratio
Enamel	84,100^a^	0.20^a^
Dentine	18,600^a^	0.31^a^
Cortical bone	13,800^a^	0.26^a^
Cancellous bone	345^a^	0.31^a^
Pulp	2^b^	0.45^b^
Stainless steel	20,0000^c^	0.30^c^

^a^Jones et al. (2001)

^b^Qian et al. (2008)

^c^Kojima and Fukui (2006)

### HG modeling

**Fig. 1:**
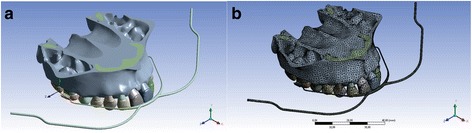
Geometry **(a)** and mesh **(b)** of the maxilla and HG, global (outside of maxilla) and local (below second molar) coordinates

To better represent clinical conditions and standardization, an HG facial bow was modeled by Solidworks® software (Fig. 1).

The HG inner bow was passively adapted to the dental arch and connected to the first molars by stainless steel tubes. These tubes were connected to the teeth in the same position as the HG band tubes (Fig. 1). As the terminal ends of the HG outer bow (hooks where elastics are attached) are mathematically unnecessary, they were disregarded; instead, the HG outer bows ended at the first molar center of resistance (Fig. 1).

This modeling was exported to ANSYS® v. 12.1 software. As the facial bow and band components are made of 18/8 stainless steel [25], their Young’s modulus was 200 GPa and the Poison’s ratio was 0.3 [26].

### Direction of HG forces

A patient who was being treated with a CP HG was selected for this study. She was 11 years and 5 months of age and presented with an angle class II, division 1 malocclusion, with permanent dentition. Cephalometrically, she exhibited predominant vertical growth (FMA = 31.0°) and a class I skeletal relationship (ANB = 1.5°). Axial and profile photographs were taken using LP, HP, and CP HGs. The sagittal, coronal, and transverse angles between each HG pull force and the occlusal plane with LP, HP, and CP HGs were measured in the photographs.

### Magnitude of HG forces

To simulate orthopedic-orthodontic forces, clinical trials with skeletal and dental class II samples of growing patients were chosen as references to determine the magnitudes of HG forces. The utilized forces on each side were 450 gf via the LP, [8], 500 gf via the HP [4], and 150 gf (LP) and 150 gf (HP) via the CP HG [5].

To simulate exclusive orthodontic forces, the necessary force to distalize a first molar was chosen; [27] the forces used on each side were 200 gf via the LP and HP and 100 gf (LP) and 100 gf (HP) via the CP HG.

### Data analysis

Using the above data, two coordinate systems were defined as follows: a “global coordinate” system, in which *x*, *y*, and *z* represented the anteroposterior, vertical, and transverse directions, respectively, and a “local coordinate” system with the same features as the “global coordinate” system, except that the “*x*” coordinate coincided with the occlusal plane (Fig. 1).

Based on these references, the tendencies of teeth movement due to LP, HP, and CP HGs, which applied orthopedic-orthodontic and exclusively orthodontic forces in the anteroposterior, vertical, and transverse planes, were calculated.

## Results

### Final model

The final model (maxilla, teeth, PDL, band tube, and HG) consisted of 434,046 elements and 578,971 nodes (Fig. 1).

### Tendencies of teeth movement

**Fig. 2:**
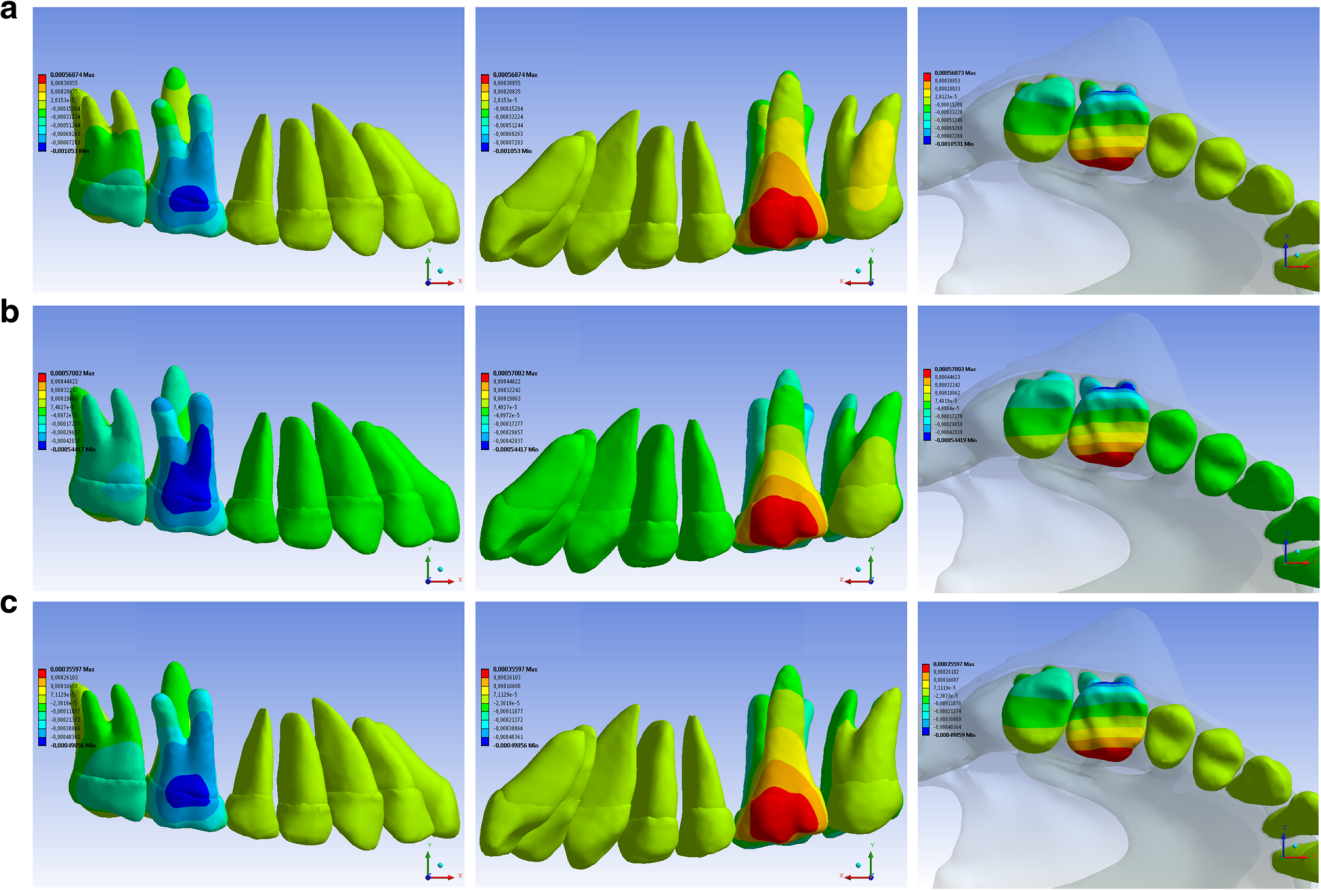
Buccal, occlusal, and lingual views of teeth movement via the LP **(a)**, HP **(b)**, and CP **(c)** HGs applying orthopedic-orthodontic forces at the *x* coordinate (*anteroposterior direction*). Exclusively orthodontic forces presented the same movement distribution and proportionately lower values. *Blue areas* represent distalization, and *red areas* represent mesialization

**Fig. 3:**
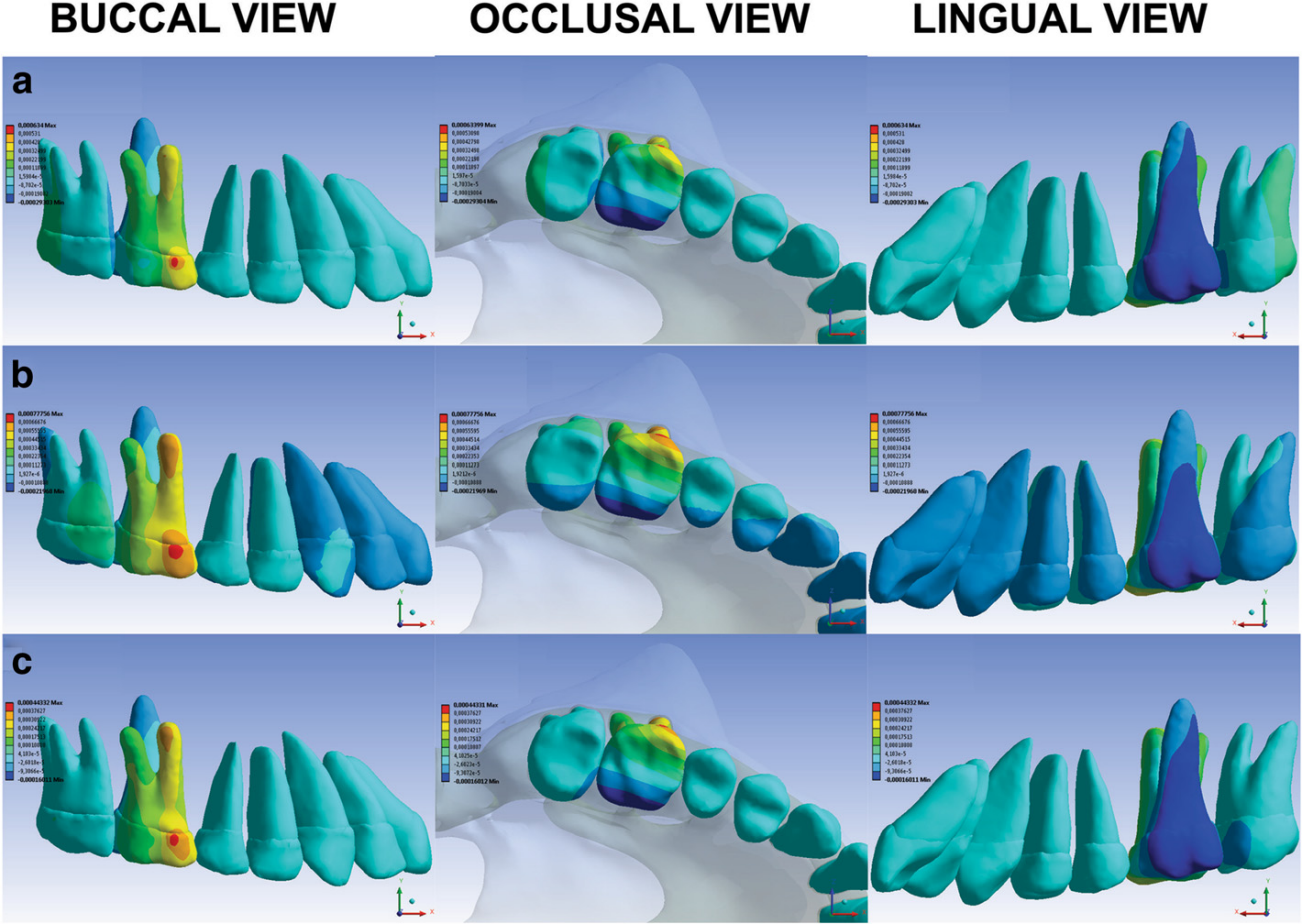
Buccal, occlusal, and lingual views of teeth movement via the LP **(a)**, HP **(b)**, and CP **(c)** HGs applying orthopedic-orthodontic forces at the *y* coordinate (*vertical direction*). Exclusively orthodontic forces presented the same movement distribution and proportionately lower values. *Blue areas* represent extrusion, and *red areas* represent intrusion

**Fig. 4:**
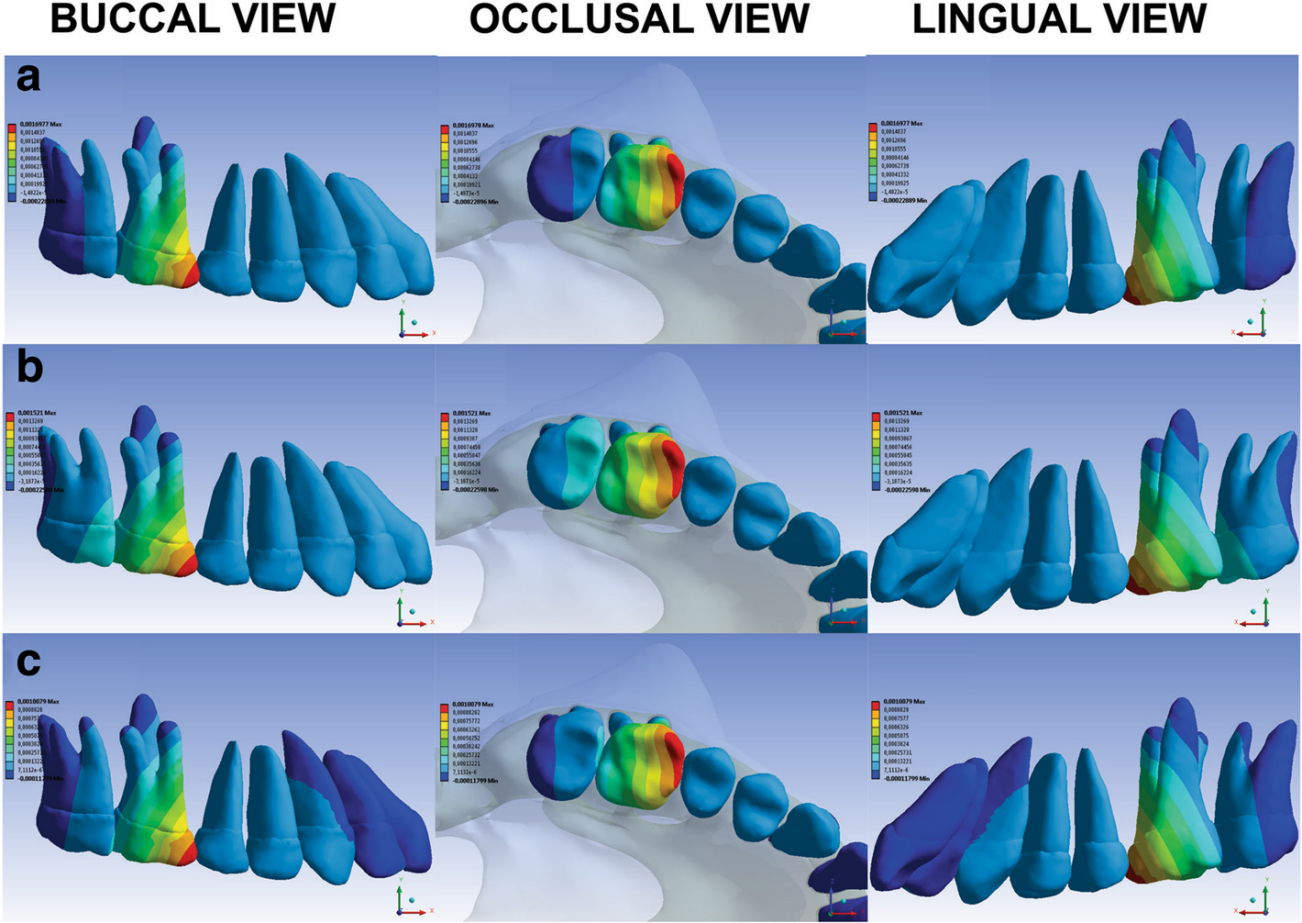
Buccal, occlusal, and lingual views of teeth movement via the LP **(a)**, HP **(b)**, and CP **(c)** HGs applying orthopedic-orthodontic forces at the *z* coordinate (*transverse direction*). Exclusively orthodontic forces presented the same movement distribution and proportionately lower values. *Blue areas* represent contraction, and *red areas* represent expansion

The tendencies of teeth movement for each scenario under the *x*, *y*, and *z* coordinates are presented in Figs. 2, 3, and 4, respectively.

It was observed that although the forces were applied only at the first molars, all maxillary teeth moved, mainly the first and the second molars; under the three pulls, when the orthopedic-orthodontic forces were reduced to exclusively orthodontic forces, the distributions of movement were similar, and the quantities of the movement were reduced by the same proportion.

### Quantification of movements of the first and second molars

**Fig. 5:**
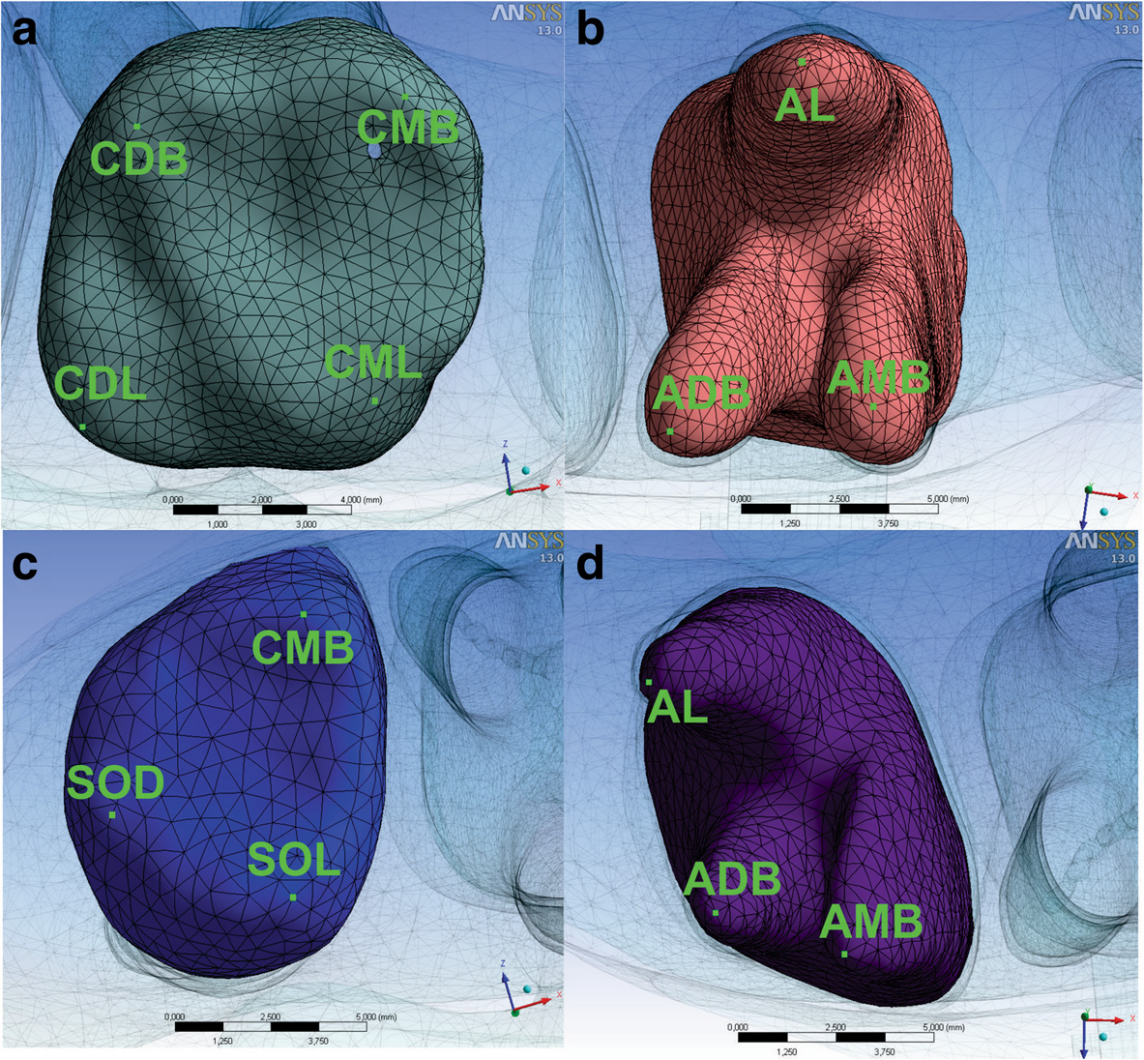
Demarcated points at the first **(a, b)** and second **(c, d)** molar crowns and roots

The first and second molars presented the greatest displacements (Table 2). Thus, to make a quantitative comparison of movements among different HG pulls and force magnitudes, the differences between them were compared. In the first molar, four crown points (Fig. 5a) representing its cusps and three root points (Fig. 5b) representing its root apices were demarcated. In the second molar, whose cusps were less defined, three crown points (Fig. 5c) representing its surfaces and three root points (Fig. 5d) representing its roots apices were demarcated.

**Table 2:** Quantification of the first and second molar movements, at anteroposterior, vertical, and transverse directions, according to the HG pull and magnitude of force

Tooth	Points	Coordinate	Quantification of movement (×10^−3^ mm)					
			Low pull		High pull		Combined pull	
			450 gf	200 gf	500 gf	200 gf	150 gf/150 gf	100 gf/100 gf
First molar	Crown							
	CMB	*x*	−0.3563	−0.1582	−0.0827	−0.0330	−0.1366	−0.0911
		*y*	0.3004	0.1335	0.5167	0.2066	0.2570	0.1713
		*z*	1.5992	0.7103	1.4753	0.5898	0.9629	0.6418
	CDB	*x*	−0.4943	−0.2195	−0.1578	−0.0631	−0.2033	−0.1355
		*y*	−0.0125	−0.0055	0.2696	0.1079	0.0815	0.0544
		*z*	0.4791	0.2130	0.6867	0.2748	0.3659	0.2440
	CML	*x*	0.4528	0.2007	0.5076	0.2027	0.3009	0.2004
		*y*	−0.1989	−0.0884	−0.0808	−0.0323	−0.0871	−0.0581
		*z*	1.1112	0.4936	1.0832	0.4331	0.6874	0.4582
	CDL	*x*	0.3127	0.1385	0.4004	0.1598	0.2236	0.1489
		*y*	−0.2456	−0.1090	−0.1718	−0.0686	−0.1305	−0.0870
		*z*	0.2066	0.0921	0.3934	0.1576	0.1886	0.1259
	Root							
	AMB	*x*	−0.3529	−0.1566	−0.2535	−0.1012	−0.1897	−0.1264
		*y*	0.3132	0.1392	0.5190	0.2076	0.2616	0.1744
		*z*	−0.0618	−0.0275	−0.1689	−0.0676	−0.0727	−0.0485
	ADB	*x*	−0.3348	−0.1490	−0.3785	−0.1516	−0.2238	−0.1493
		*y*	0.1648	0.0732	0.2564	0.1025	0.1323	0.0882
		*z*	0.0102	0.0045	−0.0099	−0.0040	0.0000	−0.0000
	AL	*x*	−0.1809	−0.0801	−0.1218	−0.0486	−0.0946	−0.0630
		*y*	−0.1643	−0.0730	−0.0611	−0.0244	−0.0702	−0.0468
		*z*	−0.0751	−0.0333	−0.1288	−0.0515	−0.0641	−0.0427
Second molar	Crown							
	CMB	*x*	−0.3817	−0.1696	−0.1041	−0.0416	−0.1514	−0.1009
		*y*	−0.0696	−0.0309	0.1015	0.0407	0.0106	0.0071
		*z*	0.0843	0.0377	0.2831	0.1134	0.1158	0.0773
	SOD	*x*	−0.2720	−0.1208	−0.0520	−0.0208	−0.1009	−0.0672
		*y*	0.0289	0.0129	0.0588	0.0235	0.0276	0.0184
		*z*	−0.1529	−0.0680	0.0610	0.0244	−0.0281	−0.0187
	SOL	*x*	−0.0768	−0.0338	0.1300	0.0522	0.0173	0.0116
		*y*	−0.0381	−0.0169	−0.0283	−0.0113	−0.0207	−0.0138
		*z*	−0.0214	−0.0094	0.1964	0.0787	0.0555	0.0371
	Root							
	AMB	*x*	−0.0434	−0.0193	−0.0856	−0.0343	−0.0406	−0.0271
		*y*	−0.0119	−0.0052	0.0722	0.0289	0.0192	0.0128
		*z*	0.0076	0.0034	−0.0001	−0.0000	0.0021	0.0014
	ADB	*x*	−0.0486	−0.0216	−0.0654	−0.0262	−0.0358	−0.0239
		*y*	0.0092	0.0041	0.0791	0.0317	0.0279	0.0186
		*z*	−0.0089	−0.0040	−0.0244	−0.0098	−0.0105	−0.0070
	AL	*x*	−0.0161	−0.0072	−0.0192	−0.0077	−0.0111	−0.0074
		*y*	−0.0044	−0.0020	−0.0106	−0.0043	−0.0047	−0.0031
		*z*	−0.0223	−0.0100	−0.0320	−0.0128	−0.0171	−0.0114

*Abbreviations*: *x coordinate* anteroposterior direction, *y coordinate* vertical direction, *z coordinate* transverse direction, *CMB* mesiobuccal cusp, *CDB* distobuccal cusp, *CML* mesiolingual cusp, *CDL* distolingual cusp, *SOD* occluso-distal surface, *SOL* occluso-lingual surface, *AMB* mesiobuccal root apex, *ADB* distobuccal root apex, *AL* lingual root apex

It was noticed that under the same pull, as the forces were reduced from orthopedic-orthodontic to exclusively orthodontic magnitudes, the quantity of movement decreased proportionally.

At the *x* coordinate, the LP HG promoted the greatest distalization. With the CP HG, the first molar presented 38.3 (CMB) to 41.1 % (CDB), and the second molar presented 37.1 (SOD) to 39.7 % (CMB) of LP HG distalization. Finally, with the HP HG, the first molar presented 23.2 (CMB) to 31.9 % (CDB), and the second molar presented 19.1 (SOD) to 27.3 % (CMB) of LP HG distalization.

At the *y* coordinate, the difference among the three pulls occurred at the first molar. Under the three pulls, similar quantities of crown and root movements were elicited, including the first molar CMB intrusion, with the HP HG presenting the greatest intrusion, followed by the LP HG (58.1 % of HP HG intrusion) and the CP HG (49.7 % of HP HG intrusion); and lingual cusp extrusion, with the LP HG presenting the greatest extrusion, followed by the HP HG, with 40.6 (CML) to 70.0 % (CDL) of LP HG extrusion, and by the CP HG, with 43.8 (CML) to 53.1 % (CDL) of LP extrusion.

CDB behavior was different among the three pulls: the LP HG promoted CDB extrusion, whereas the HP HG and CP HG promoted CDB intrusion (CP HG intrusion was 30.2 % of HP HG intrusion).

At the *z* coordinate, the three pulls promoted similar first molar behavior and different second molar behavior. At the first molar, the three pulls promoted expansion of the mesial cusps, contraction of the distal cusps, and limited movements at the root apices.

The LP HG presented the greatest mesial surface expansion of the first molar, followed by the HP HG, with 92.3 (CMB) to 97.5 % (CML) of LP HG expansion, and by the CB HG, with 60.2 (CMB) to 61.9 % (CML) of LP expansion.

The HP HG presented the greatest contraction of the distal surface of the first molar, followed by the LP HG, with 52.5 (CDL) to 69.8 % (CDB) of HP HG contraction, and by the CP HG, with 47.9 (CDL) to 53.3 % (CDB) of HP contraction.

Regarding the second molar, the LP HG contracted the distal surface of the crown and facilitated limited movement of the remainder of the tooth crown and roots. The HP HG expanded the mesiobuccal and lingual crown surfaces and elicited limited movement of the remainder of the tooth crown and roots. The CP HG expanded the mesiobuccal crown surface and elicited limited movement of the remainder of the tooth crown and roots.

## Discussion

### Methodology

Previous investigations of HGs utilizing FEA have focused on skeletal effects and modeled only the bone and the first molar [21], simplified the directions of the forces [21], or assumed the isotropic and linearly elastic behavior of the PDL [28]. Our study tried to overcome these limitations by not only modeling the maxilla, the maxillary teeth, and the HG but also by respecting the viscoelastic behavior of the PDL.

Although the PDL exhibits non-linear behavior [29], some studies have assumed that the PDL exhibits linear behavior [22]. Toms and Eberhardt [22] demonstrated that different stresses are obtained when linear or non-linear behavior of the PDL is assumed. Our study utilized the criteria of Natali et al. [24], who developed a constitutive model considering the fluid fluxes and internal conformational rearrangements of the collagen and elastin of the PDL to represent the typical nonlinear and hyperelastic mechanical behavior of the PDL.

### Tendencies of teeth movement

In the transverse direction (*z* coordinate), the three HG pulls tended to expand the first molar and to contract the second molar (Fig. 4 and Table 2). The tendency of the first molar expansion also occurs in vivo [30]. With the LP HG, all distal and lingual surfaces of the second molar contracted. With the CP HG, the distal surface of the second molar contracted. With the HP HG, the distal portions of the lingual and distobuccal roots of the second molar contracted. These results suggest that the HG inner bow should be expanded during activations, independent of the pull that is utilized.

Data from LP HG-related clinical trials recommend 4 to 8 mm [8] and 10 mm [31] of HG inner bow expansion to overcome the tendency toward contraction and to expand the dental arches. With the HP HG, Firouz et al. [4] utilized a transpalatal arch to maintain symmetry and arch width and to prevent molar rotation. With the CP HG, some authors [5] did not cite any procedures controlling the transverse effects of the HG.

In the anteroposterior direction (*x* coordinate), the three pulls promoted rotation of the first and second molars as their buccal surface moved distally, and their lingual surface moved mesially (Fig. 2 and Table 2). This type of movement cannot be evaluated in studies that utilize cephalometry. Regarding the distalization tendencies of the first and second molars, the LP HG presented the greatest values, with the crown moving more than the roots, i.e., showing a tendency toward tipping rather than translatory movement. The CP HG and the HP HG have less tendency to move the molars distally but promote more translatory movement than tipping movement.

With the LP HG, it was interesting to observe that distal tipping of the first and second molars occurred even while applying the HG force at the trifurcation of the first molar roots, which is the center of resistance for this tooth [7]. One reason for this phenomenon may be the deformation of the HG outer bow caused by force application [30]. This deformation may move the force application line downwards.

Our study utilized the same length and inclination of the HG outer bow and the same magnitude of force used by Firouz et al. [4] These authors showed that the HP HG promotes first molar distalization and that the roots moved more than the crown, which is the same tendency of movement observed in our study.

With the CP HG, we observed that the first molar tends to distalize in a translatory movement and that the second molar tends to distalize in a tipping movement. In a previous clinical trial [5], distalization and tipping of the second premolar as well as the first and second molars occurred.

Our study results are consistent with the data obtained by Baumrind et al. [32], who considered only translatory distalization a success and showed that the incidence of successful distal displacement with each pull was as follows: LP HG—33.9 %, HP HG—71.7 %, and CP HG—71.4 %. In contrast, O’Reilly et al. [33] did not find differences between HP and LP distalization and showed that both pulls promoted first molar tipping.

In the vertical direction (*y* coordinate) (Fig. 3 and Table 2), the tendency of first molar extrusion with the LP HG [34] was confirmed by greater values of lingual cusp extrusion, as well as by the tendency toward distobuccal cusp extrusion, as the other pulls promote first molar intrusion.

With the HP HG, the extrusion values were lower than those associated with the LP HG and CP HG. Additionally, the values of first molar distobuccal cusp intrusion were higher than those of the CP HG. This confirms the results of a clinical trial [4] showing that the HP HG tends to intrude and distalize the first molars.

Our study verified that the CP HG tends to promote similar types of vertical effects to those of the HP HG but with lower values. In a previous clinical trial [5], when the CP HG was utilized, the first and second molars exhibited only limited movement in the vertical direction, even with significant distalization.

When the forces applied by HGs are reduced, their orthopedic effects are also reduced [31]. For the three HG pulls, when the orthopedic-orthodontic forces were reduced to exclusively orthodontic forces, the distributions of teeth movement were maintained, and their values reduced by the same proportion. Thus, the distribution of teeth movement depends on the direction of HG pull and not on the magnitude of applied force.

### Clinical implications

FEA of tooth movement represents only the tendency of displacement before bone remodeling. Structural changes in bone and in periodontal supporting tissues during teeth movement lead to changes in their biomechanical behavior and, consequently, to modifications of local stresses and strains [35].

## Conclusions

In the model utilized for this study, the simulations of headgear action generated the following tendencies:

Regarding teeth movement

1. The LP HG promoted the greatest distalization, followed by the CP HG and HP HG.
2. The LP HG extruded the first molar lingual and distobuccal cusps.
3. The HP HG intruded the buccal cusps of the first molar and, compared to the LP HG, promoted less extrusion of the first molar lingual cusps.
4. The CP HG promoted similar vertical effects to those of the HP but with lower values.
5. With the LP HG, there was contraction of the lingual and distal surfaces of the second molar.
6. With the HP and CP, there was contraction of the lingual and distobuccal roots of the second molar.

Regarding magnitude of forces

1. With the same headgear pull, when orthopedic-orthodontic forces were reduced to exclusively orthodontic forces, the distribution of movements was maintained, and the values were reduced by the same proportion.
